# Supplementary material for: Advanced lipodystrophy reverses fatty liver in mice lacking adipocyte hormone-sensitive lipase
Source: Commun Biol. 2021 Mar 10;4:323. doi: 10.1038/s42003-021-01858-z (PMC7946939; doi:10.1038/s42003-021-01858-z)
Supplement: Supplementary file 2 — Supplementary Information [file 42003_2021_1858_MOESM2_ESM.pdf]

Supplementary information

Advanced lipodystrophy reverses fatty liver in mice lacking adipocyte hormone-sensitive lipase

Laura Pajed[1*], Ulrike Taschler[1*], Anna Tilp[1], Peter Hofer[1], Petra Kotzbeck[2], Stephanie Kolleritsch[1], Franz P.W. Radner[1], Isabella Pototschnig[1], Carina Wagner[1], Margarita Schratter[1], Sandra Eder[1], Sabrina Huetter[1], Renate Schreiber[1], Guenter Haemmerle[1], Thomas O. Eichmann[1,3], Martina Schweiger[1], Gerald Hoefler[4,5], Erin E. Kershaw[6], Achim Lass[1,5], and Gabriele Schoiswohl[1#]

[1]Institute of Molecular Biosciences, University of Graz, Heinrichstraße 31/II, 8010 Graz, Austria

[2]Division of Endocrinology and Diabetology, Department of Internal Medicine, Medical University of Graz, Auenbruggerplatz 15, 8036 Graz, Austria

[3]Center for Explorative Lipidomics, BioTechMed-Graz, Humboldtstraße 50, 8010 Graz, Austria

[4]Diagnostic & Research Institute of Pathology, Medical University of Graz, Neue Stiftingtalstraße 6, 8010 Graz, Austria

[5]BioTechMed Graz, 8010 Graz, Austria

[6]Department of Medicine, University of Pittsburgh School of Medicine, 200 Lothrop Street, Pittsburgh, PA 15213, USA

SUPPLEMENTAL TABLES

Suppl Table 1: Antibody Table

Protein	Protein name	Company	Dilution used
ABHD5	Alpha/beta hydrolase domain containing 5	Abnova, H00051099-M01	1:1, 000 in 5% milk
ACC	Acetlyl coenzyme A carboxylase	Cell Signaling, 3676	1:20,000 in 5% milk
AKT	RAC-alpha serine/threonine-protein kinase	Cell Signaling, C67E7	1:1,000 in 5% BSA
ATGL	Adipose triglyceride lipase	Cell signaling, 2138S	1:1,000 in 5% milk
FAS	Fatty acid synthase	Cell Signaling, 3180	1:6,000 in 5% milk
GAPDH	Glyceraldehyde-3-phosphate dehydrogenase	Cell signaling, 2118S	1:10,000 in 5% milk
HSL	Hormone-sensitive lipase	Cell signaling, 4107S	1:5,000 in 5% milk
PLIN1	Perilipin-1	Sigma-Aldrich, P1998	1:1,000 in 5% milk
pAKT (Ser473)	RAC-alpha serine/threonine-protein kinase	Cell Signaling, 9271S	1:1,000 in 5% BSA
SCD1	Stearoyl-CoA desaturase	Cell Signaling, 2438	1:1,000 in 5% milk
SREBP1c	Sterol regulatory element-binding protein 1; clone 2A4	Abcam, ab3259	1:500 in 1% milk

Suppl Table 2: Primer sequences used for quantitative gene expression analysis

Gene	Gene name	Primer sequence
Acc1	Acetyl-CoA carboxylase 1	F: 5'- GCCAGCTCTGGAGGTGTATG -3'<br>R: 5'- TCCTTAAGCTGGCGGTGTT -3'
Cd11c	Cluster of differentiation 11c	F: 5'- CAGTGACCCCGATCACTCTT -3'<br>R: 5'- CACCACCAGGGTCTTCAAGT -3'
Cd36	Cluster of differentiation 36	F: 5'- GAACCTATTGAAGGCTTACATCC- 3'<br>R: 5'- CCCAGTCACTTGTGTTTTGAAC- 3'
Cebp1a	CCAAT enhancer binding protein alpha	F: 5'- CAAGAACAGCAACGAGTACCG -3'<br>R: 5'- GTCACTGGTCAACTCCAGCAC -3'
Chrebp	Carbohydrate responsive element-binding protein	F: 5'- CTGGGGACCTAAACAGGAGC -3'<br>R: 5'- GAAGCCACCCTATAGCTCC -3'
Cpt1a	Carnitine palmitoyltransferase 1A	F: 5'- CACCAACGGGCTCATCTTCTA -3'<br>R: 5'- CAAAATGACCTAGCCTTCTATCGAA -3'
Col1a1	Collagen type I alpha 1	F: 5'- CCGGCTCCTGCTCCTCCTA -3'<br>R: 5'- CCATTGTGTATGCAGCTGACTTC -3'
Col1a2	Collagen type I alpha 2	F: 5'- AAGGGTGCTACTGGACTCCC -3'<br>R: 5'- TTGTTACCGGATTCTCCTTTGG -3'
Cyclo B	Cyclophilin B	F: 5'- GGCTCCGTCGTCTTCCTTTT -3'<br>R: 5'- ACTCGTCCTACAGATTCATCTCC -3'
Dgat2	Diacylglycerol acyltransferase 2	F: 5'- TTCCTGGCATAAGGCCCTATT -3'<br>R: 5'- AGTCTATGGTGTCTCGGTTGAC -3'
Dio2	Deiodinase type II	F: 5'- CAGTGTGGTGCACGTCTCCAATC -3'<br>R: 5'- TGAACCAAAGTTGACCACCAG -3'
Fabp1	Fatty acid binding protein 1	F:5'- TGCCACCATGAACTTCTCCGGC -3'<br>R:5'- TCCAGTTCGCACTCCTCCCCC -3'
Fabp3	Fatty acid binding protein 3	F: 5'- CCCCTCAGCTCAGCACCAT -3'<br>R: 5'- CAGAAAAATCCCAACCCAAGAAT -3'
Fabp4	Fatty acid binding protein 4	F: 5' - GAACCTGAAGCTTGTCTTCG -3'<br>R: 5' ACCAGCTTGTCACCATCTCG -3'
Fasn	Fatty acid synthase	F: 5'- TCCTGGAACGAGAACACGATCT -3'<br>R: 5'- GAGACGTGTCACTCCTGGACTTG -3'
F4/80	Adhesion G protein-coupled receptor E1	F: 5'- GGATGTACAGATGGGGGATG -3'<br>R: 5'- CATAAGCTGGGCAAGTGGTA -3'
Gck	Glucokinase	F: 5'- TGGTGGATGAGAGCTCAGTG -3'<br>R: 5'- TGAGCAGCACAAGTCGTACC -3'
Glut2	Glucose transporter 2	F: 5'- TTCCAGTTCGGCTATGACATCG -3'<br>R: 5'- CTGGTGTGACTGTAAGTGGGG -3'
Gyk	Glycerol kinase	F: 5'- GAGCTCTTTTTGGGACCATT -3'<br>R: 5'- AGGCCATAGATCTCAGAAGA -3'
G6Pase	Glucose 6-phosphatase	F: 5'- CCTCCTCAGCCTATGTCTGC -3'<br>R: 5'- AACATCGGAGTGACCTTTGG -3'
Hsl	Hormone-sensitive lipase	F: 5'- GCTGGGCTGTCAAGCACTGT -3'<br>R: 5'- GTAACTGGGTAGGCTGCCAT -3'
Lcad	Acyl-Coenzyme A dehydrogenase, long-chain	F: 5'- CCGGTTCTTTGAGGAAGTGAA -3'<br>R: 5'- AGTGTCGTCCTCCACCTTCTC -3'
Lxra	Liver X receptor alpha	F: 5'- AGGAGTGTCGACTTCGCAAA -3'<br>R: 5'- CTCTTCTTGCCGCTTCAGTTT -3'
Pepck	Phosphoenolpyruvate carboxykinase	F: 5'- CATATGCTGATCCTGGGCATAAC -3'<br>R: 5'- CAAACTTCATCCAGGCAATGTC -3'
Pgc1a	Peroxisome proliferative-activated receptor gamma coactivator 1 alpha	F: 5'- CCCTGCCATTGTTAAGACC -3'<br>R: 5'- TGCTGCTGTTCCTGTTTTC -3'
Ppara	Peroxisome proliferator-activated receptor alpha	F: 5'- GTACCACTACGGAGTTCACGCAT -3'<br>R: 5'- CGCCGAAAGAAGCCCTTAC -3'
Pparg2	Peroxisome proliferator-activated receptor gamma 2	F: 5'- CCAGAGCATGGTGCCTTCGCT -3'<br>R: 5'- CAGCAACCATTGGGTCAG -3'
Prdm16	PR domain-containing protein 16	F: 5'- CAGCACGGTGAAGCCATTC -3'<br>R: 5'-GCG TGC ATC CGC TTG TG-3'
Scd1	Stearoyl-CoA desaturase-1	F: 5'- CCTGCGGATCTTCCTTATCATT -3'<br>R: 5'- GTGGGCGCGGTGATCTC -3'
Srebp1c	Sterol regulatory element-binding protein 1c	F: 5'- GTTACTCGAGCCTGCCTTCAGG -3'<br>R: 5'- CAAGCTTTGGACCTGGGTGTG -3'
Tgfb	Transforming Growth Factor beta	F: 5'- CACCGGAGAGCCCTGGATA -3'<br>R: 5'- TGTACAGCTGCCGCACACA -3'
Ucp1	Uncoupling protein 1	F: 5'- ACTGCCACACCTCCAGTCATT -3'<br>R: 5'- CTTTGCCTCACTCAGGATTGG -3'

SUPPLEMENTARY FIGURES

Supplementary Fig. 1

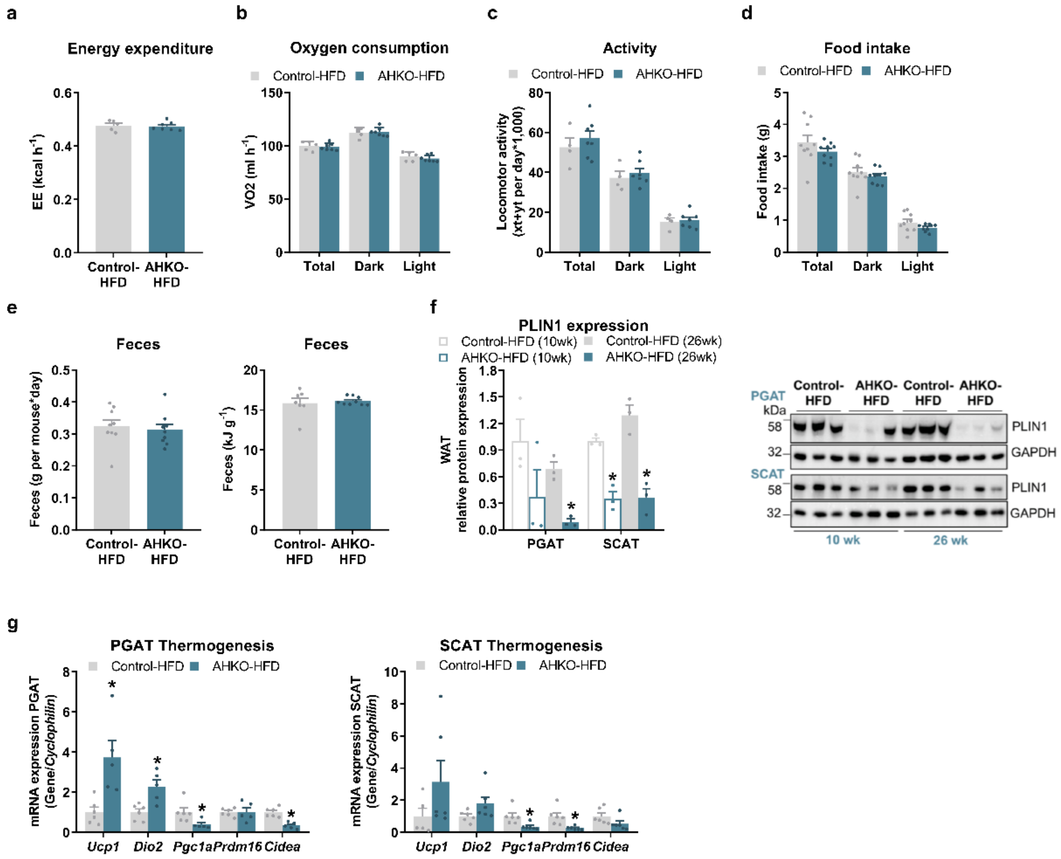

Supplementary Fig. 1. Effects of adipocyte-specific HSL deletion on energy homeostasis of mice fed HFD.

a-c. Metabolic phenotyping of mice using a laboratory animal monitoring system (M, 24wk, HFD, *ad libitum* fed, n=5-7 animals/genotype). **a.** Energy expenditure (EE) per day, calculated using the formula: heat x 0,859 845 227 858 99 kcal/h (1 Watt), **b.** oxygen consumption, and **c.** locomotor activity. **d.** Food intake of single-housed mice per day (M, 30wk, HFD, *ad libitum* fed, n=4-9 animals/genotype). **e.** Left: Feces output was determined on 5 consecutive days. Right: Feces were collected on 5 consecutive days, pooled, and analyzed for the excreted energy using bomb calorimetry (M, 30wk, HFD, *ad libitum* fed, n=7-9 animals/genotype). **f.** Left panel: Perilipin1 (PLIN1) protein expression in PGAT and SCAT. Right panel: Representative immunoblots (M, 10wk & 26wk, HFD, *ad libitum* fed, n=3 animals/genotype). **g.** PGAT (left) and SCAT (right) mRNA expression of genes involved in thermogenesis relative to *Cyclophilin* reference gene by qPCR with control animals arbitrarily set to 1

for each gene (M, 24wk, HFD, *ad libitum* fed, n=5-6 animals/genotype). Data represent mean + SEM. Statistical significance was determined by Student's two-tailed *t*-test. $P < 0.05$: * for effect of genotype.

Supplementary Fig. 2

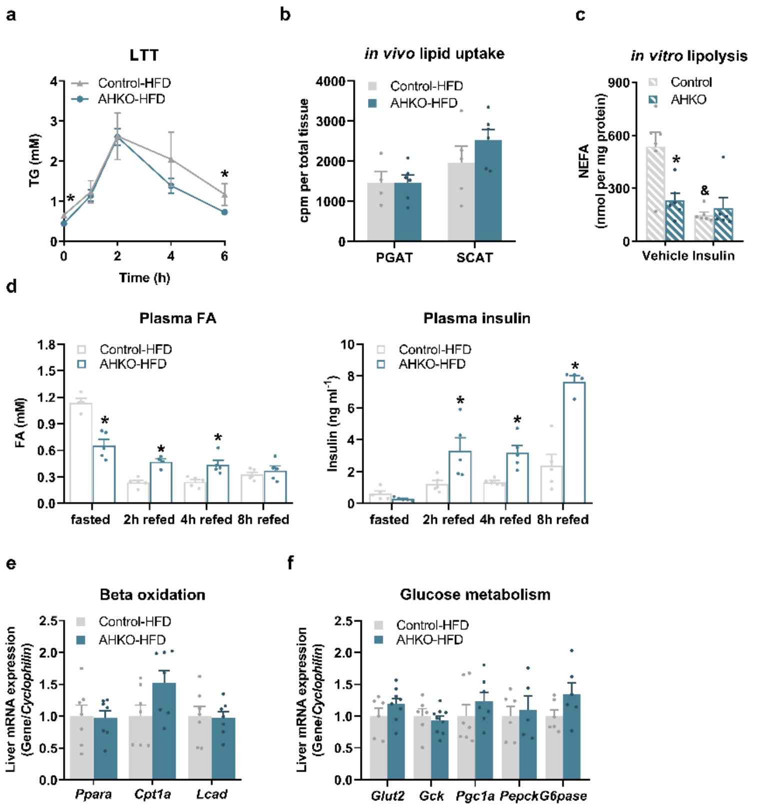

Supplementary Fig. 2: Effects of adipocyte-specific HSL deletion on systemic and tissue-specific lipid and glucose metabolism.

a. Lipid tolerance test (LTT) was performed by oral olive oil gavage and subsequent measurement of plasma triglycerides (TG; M, 26wk, HFD, 4h-fasted, n=5-9 animals/genotype). **b.** *In vivo* lipid uptake into WAT 2h after of an oral olive oil gavage containing [^{3}H]-labeled triolein (M, 20wk, HFD, 12h-fasted, n=5-6 animals/genotype). **c.** *In vitro* lipolysis determined by fatty acid (FA) release from primary adipocytes incubated without (vehicle) or with 10 ng insulin/ml in the presence of 10 nM isoproterenol for 1h. Stromal vascular fraction was isolated from SCAT and differentiation was initiated 2 days after preadipocytes reached confluency. **d.** 12h-fasted and refed plasma fatty acid (left) and insulin (right) levels at indicated time-points (M, HFD, 10wk, n=4-5 animals/genotype). **e.** Liver mRNA expression of genes involved in beta-oxidation and **f.** glucose metabolism relative to *Cyclophilin* reference gene by qPCR with control animals arbitrarily set to 1 for each gene (M, 26wk, HFD, *ad libitum* fed, n=6-9

animals/genotype). Data represent mean ± SEM. Statistical significance was determined by Student's two-tailed *t*-test. $P < 0.05$: * for effect of genotype; [&] for effect of treatment.

Supplementary Fig. 3

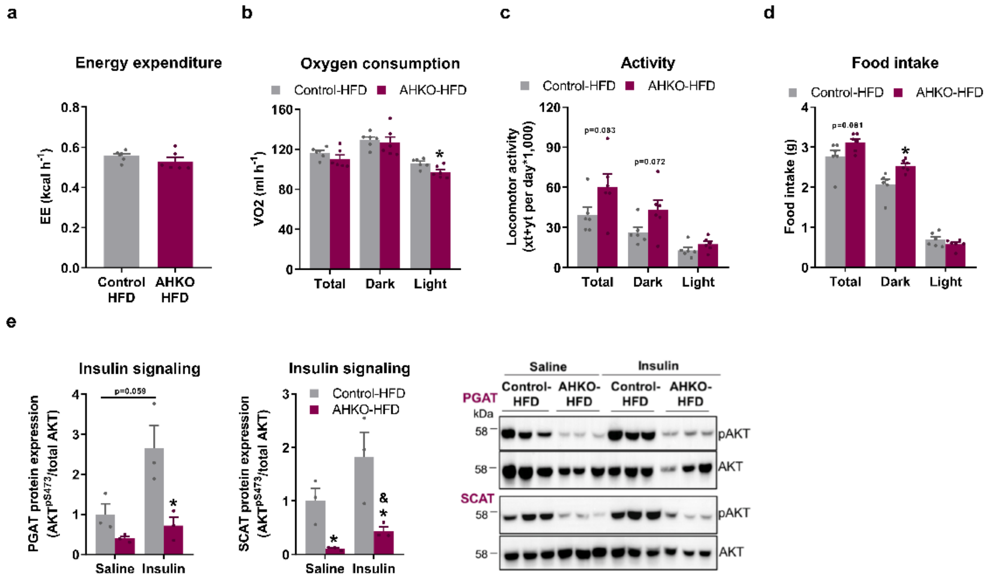

Supplementary Fig. 3: Effects of longitudinal adipocyte-specific HSL deletion on systemic energy metabolism and adipose tissue lipid and insulin signaling.

a-c. Metabolic phenotyping of mice using a laboratory animal monitoring system (M, 44wk, HFD, *ad libitum* fed, n=6 animals/genotype). **a.** Energy expenditure (EE) per day was calculated using the formula: heat x 0,859 845 227 858 99 kcal/h (1 Watt), **b.** oxygen consumption, and **c.** locomotor activity. **d.** Food intake of single-housed mice per day (M, 42wk, HFD, *ad libitum* fed, n=6 animals/genotype). **e.** Insulin signaling in WAT. Mice were injected with saline or insulin at 0.75 IU kg^{-1} body weight. Left: Quantification of phosphorylation of $AKT^{pSer473}$/total AKT in PGAT and SCAT (M, 44wk, HFD, 12h-fasted, n=3 animals/genotype). Right: Representative immunoblots. Data represent mean + SEM. Statistical significance was determined by Student's two-tailed *t*-test. $P < 0.05$: * for effect of genotype; & for effect of treatment.

Supplementary Fig. 4

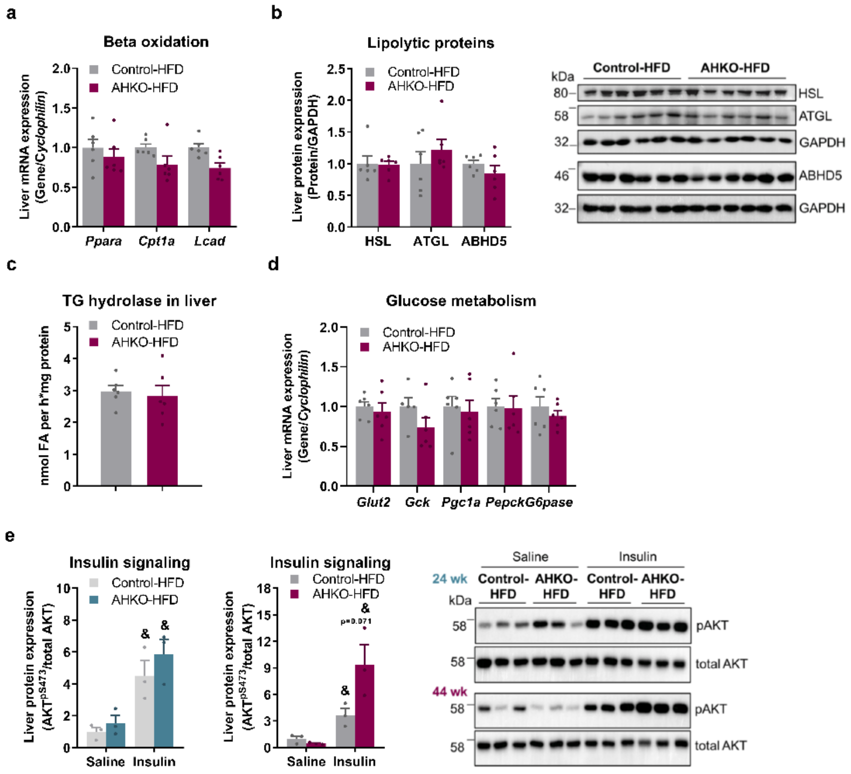

Supplementary Fig. 4: Effects of longitudinal adipocyte-specific HSL deletion on liver lipid / glucose metabolism and insulin signaling.

a. Hepatic mRNA expression of genes involved in beta-oxidation relative to *Cyclophilin* reference gene by qPCR with control animals arbitrarily set to 1 for each gene (M, 44wk, HFD, *ad libitum* fed, n=6 animals/genotype). **b.** Relative protein expression of HSL, ATGL, and ABHD5 in the liver and representative immunoblots (M, 44wk, HFD, *ad libitum* fed, n=6 animals/genotype). **c.** Triglyceride (TG) hydrolase activity in liver determined by [^{3}H]-labeled oleic acid release (M, 44wk, HFD, *ad libitum* fed, n=6 animals/genotype). **d.** Hepatic mRNA expression of genes involved in glucose metabolism relative to *Cyclophilin* reference gene by qPCR with control animals arbitrarily set to 1 for each gene (M, 44wk, HFD, *ad libitum* fed, n=5-6 animals/genotype). **e.** Insulin signaling in liver. Mice were injected with saline or insulin at 0.75 IU kg^{-1} body weight (M, 24wk & 44wk, HFD, 12h-fasted, n=3 animals/genotype). Left: Quantification of phosphorylation of AKT^{pSer473}/total AKT in PGAT and SCAT. Right: Representative immunoblots. Data represent mean + SEM. Statistical significance was determined by Student's two-

tailed *t*-test. *P* values compare effect of genotype; $P < 0.05$: * for effect of genotype; $^{\&}$ for effect of treatment.

.

Supplementary Fig. 5

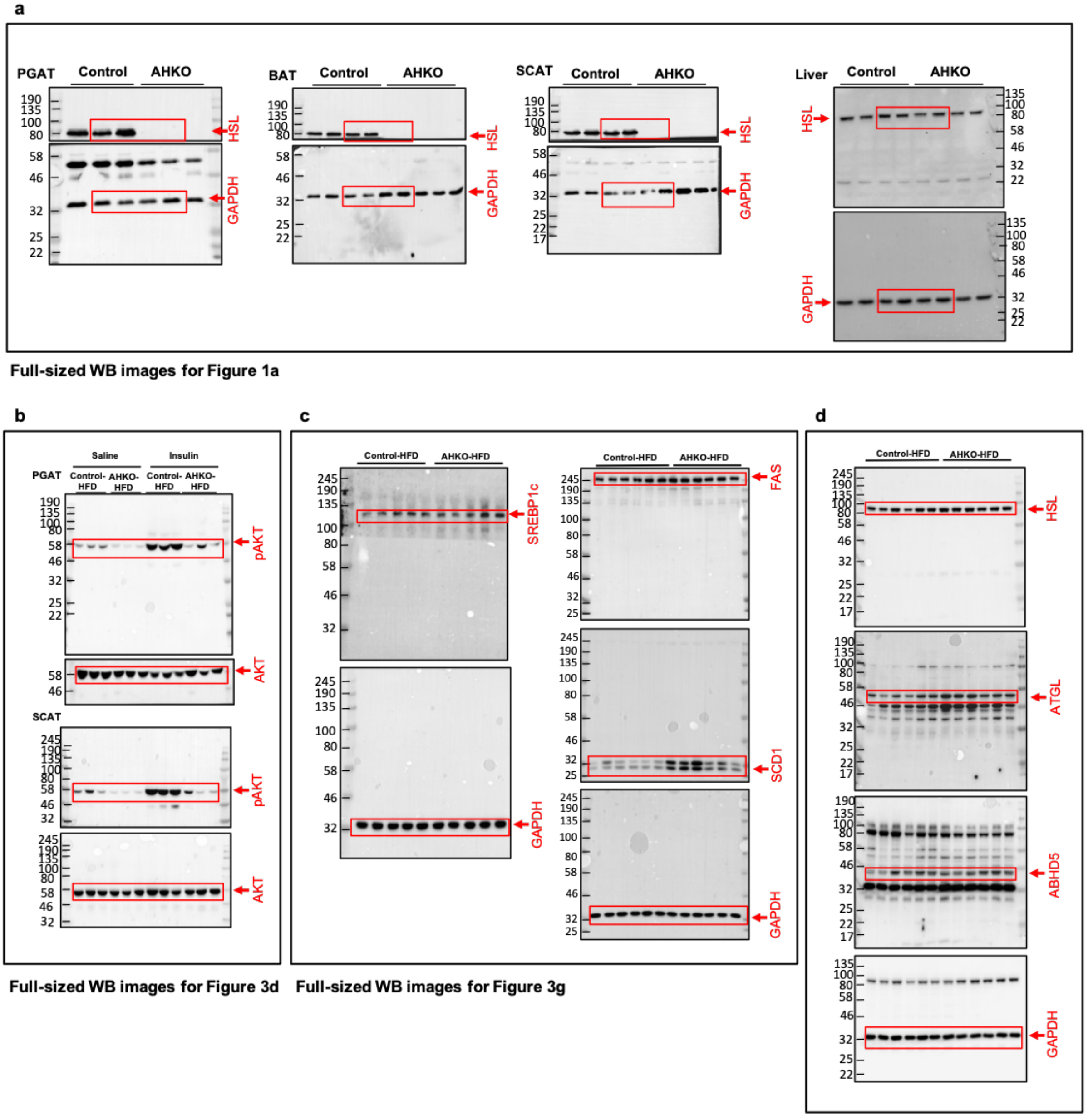

Full-sized WB images for Figure 1a

Full-sized WB images for Figure 3d

Full-sized WB images for Figure 3g

Full-sized WB images for Figure 3h

Supplementary Fig. 5: Full scan of Western blots.

a-d. Full-sized images of Western blot from Figure 1a (**a**), 3d (**b**), 3g (**c**), and 3h (**d**). Red boxes highlight areas that were displayed in the indicated figures.

Supplementary Fig. 6

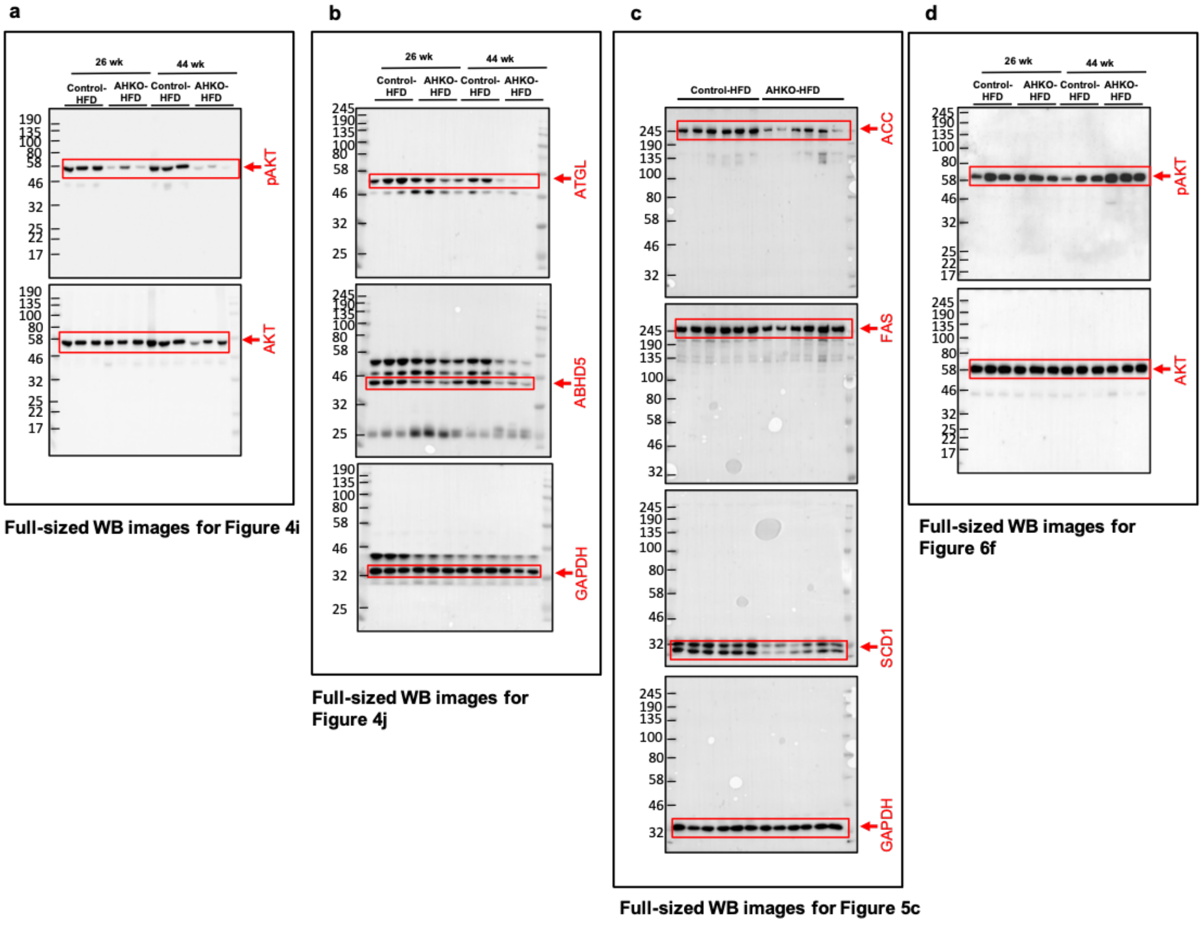

Supplementary Fig. 6: Full scan of Western blots.

a-d. Full-sized images of Western blot from Figure 4i (**a**), 4j (**b**), 5c (**c**), and 6f (**d**). Red boxes highlight areas that were displayed in the indicated figures.

Supplementary Fig. 7

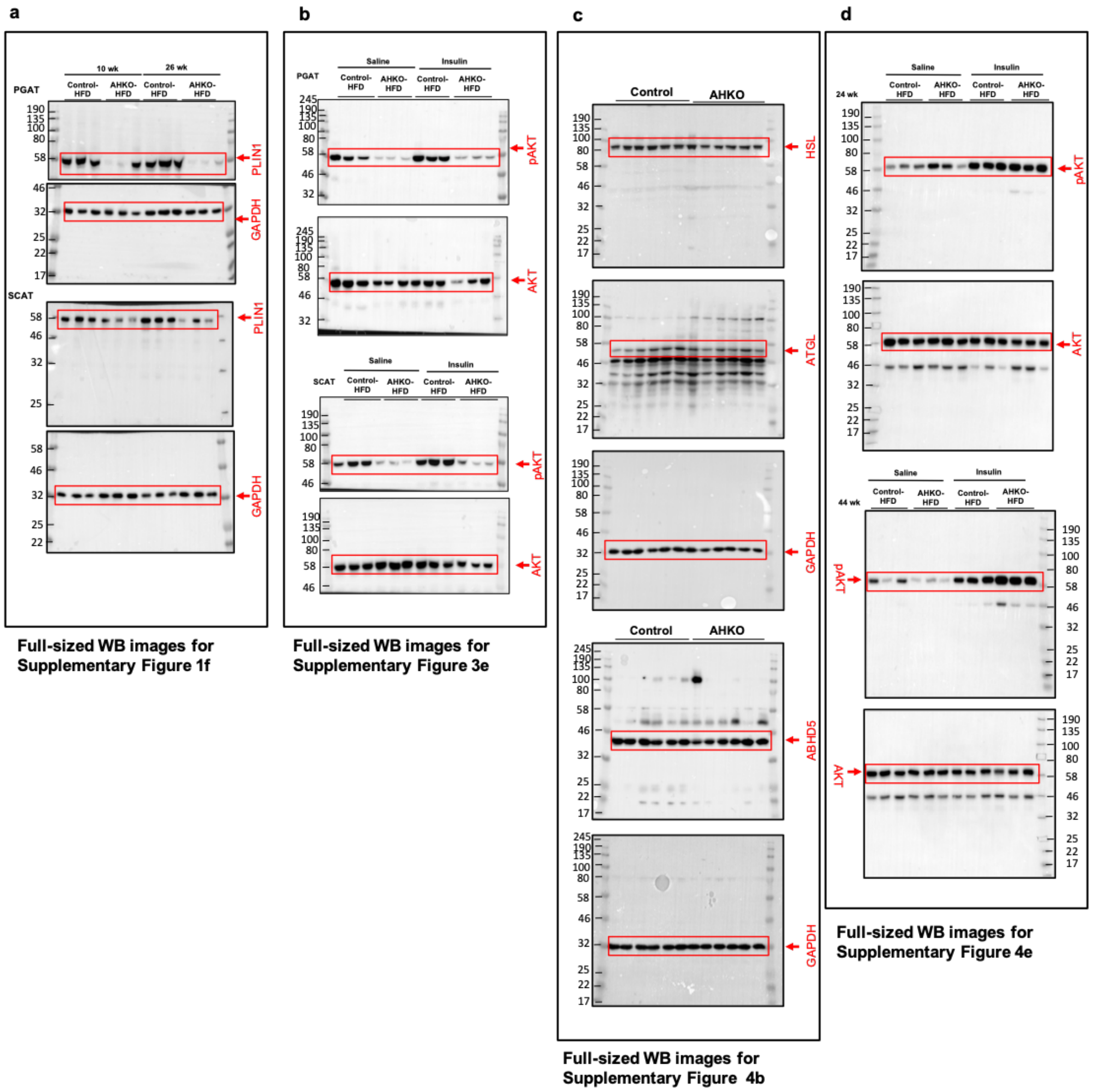

Supplementary Fig. 7: Full scan of Western blots.

a-d. Full-sized images of Western blot from Supplementary Figure 1f (**a**), 3e (**b**), 4b (**c**), and 4e (**d**). Red boxes highlight areas that were displayed in the indicated figures.

SUPPLEMENTARY METHODS

Metabolic phenotyping. For daily food intake mice were single-housed for one week and food intake was monitored for light and dark cycle. EE, oxygen consumption rate, and locomotor activity were monitored by using a laboratory animal monitoring system (PhenoMaster, TSE Systems GmbH, Bad Homburg, Germany). Before metabolic phenotyping, mice were familiarized to drinking flasks and single housing for at least 24 h. Data were separated on the basis of light:dark cycle (average of three light:dark cycles). For fecal analysis, feces of single-housed mice were sampled on 5 consecutive days and analyzed for the excreted energy using a bomb calorimeter. For the analysis, 0.9 g grounded feces were burned in an adiabatic oxygen bomb calorimeter C 200 (IKA Analysentechnik, Germany). For lipid tolerance test (LTT), mice received a gavage containing 250 µl of olive oil following a 4 h fast. Plasma triglycerides were determined using Infinity Triglycerides Liquid Stable Reagent (ThermoFisher Scientific).

***In vivo* lipid uptake.** For *in vivo* lipid uptake, 12 h-fasted mice were given 2 µCi [^{3}H]-triolein (American Radiolabeled Chemicals) in a 200 µl olive oil gavage and sacrificed 2 h after lipid bolus administration. Mice were perfused with ice-cold PBS via the aorta and tissues were collected. Tissues were lysed with 0.1% SDS/0.3 N NaOH and an aliquot of tissue lysate was used to measure the total radioactivity of [^{3}H]-triolein. Protein content was determined by Pierce™ BCA Protein Assay Kit (ThermoFisher Scientific) according to manufacturer's instructions using BSA as standard.

Isolation and differentiation of stromal vascular fraction. For isolation and differentiation of the adipose stromal vascular fraction (SVF), subcutaneous adipose tissue (SCAT) from two mice aged 10-16 weeks was dissected, washed, minced, and digested at 37°C for 45 min in PBS containing 1.5 mg/ml collagenase D (corresponds to 0.285 IU/mL; Roche Diagnostics), 3.2 mM $CaCl_2$, 15 mM HEPES, and 0.5% BSA. Upon digestion, complete DMEM/F12 media containing GlutaMAX (Life Technologies), 10% FCS (Gibco, Life Technologies), pen/strep (Gibco, Life Technologies), and primocin (Lonza Cologne) were added to the mixture and then filtered through a 100 µm cell strainer to remove undigested tissue. The flow-through was centrifuged for 5 min at 600 × *g*. Floating lipids, adipocytes, and the supernatant

were aspirated. The remaining cell pellet containing the SVF was resuspended in 1 ml red blood cell lysis buffer (154 mM NH_4Cl, 10 mM $KHCO_3$, 0.1 mM EDTA, pH 7.4) for 2 min and then mixed with complete DMEM/F12, filtered through a 40 µm cell strainer, and centrifuged as described above. Finally, the SVF was resuspended in complete media and seeded on a 10 cm cell culture dish. Differentiation was induced 2 days post-confluent by using complete DMEM/F12 supplemented with 1 µM dexamethasone, 0.5 mM isobutylmethylxanthine, 0.87 µM insulin, and 1 µM rosiglitazone. Two days after induction, cells were maintained in complete DMEM/F12 containing 0.87 µM insulin until complete differentiation at day 10.

Lipolytic activities of primary adipocytes. For *in vitro* lipolysis assay, primary adipocytes were incubated in DMEM/F12 supplemented with 2% FA-free BSA and 10 nM isoproterenol for 60 min at 37°C. The assay was performed in the presence or absence of 10 ng/ml insulin. FA release was measured using commercial kit HR Series NEFA-HR Reagents.
