# Supplementary material for: Advanced lipodystrophy reverses fatty liver in mice lacking adipocyte hormone-sensitive lipase
Source: Commun Biol. 2021 Mar 10;4:323. doi: 10.1038/s42003-021-01858-z (PMC7946939; doi:10.1038/s42003-021-01858-z)
Supplement: Supplementary file 3 — Description of Additional Supplementary Files [file 42003_2021_1858_MOESM3_ESM.pdf]

Description of Additional Supplementary Files

File Name: Supplementary Data 1

Description: All source data underlying the charts and graphs in the main table and figures.
