# Supplementary material for: Advanced lipodystrophy reverses fatty liver in mice lacking adipocyte hormone-sensitive lipase
Source: Commun Biol. 2021 Mar 10;4:323. doi: 10.1038/s42003-021-01858-z (PMC7946939; doi:10.1038/s42003-021-01858-z)
Supplement: Supplementary file 5 — Reporting Summary [file 42003_2021_1858_MOESM5_ESM.pdf]

nature research

Corresponding author(s): Gabriele Schoiswohl

Last updated by author(s): 29/1/2021

Reporting Summary

Nature Research wishes to improve the reproducibility of the work that we publish. This form provides structure for consistency and transparency in reporting. For further information on Nature Research policies, see Authors & Referees and the Editorial Policy Checklist .

Statistics

For all statistical analyses, confirm that the following items are present in the figure legend, table legend, main text, or Methods section.

n/a	Confirmed	
☐	☒	The exact sample size (*n*) for each experimental group/condition, given as a discrete number and unit of measurement
☐	☒	A statement on whether measurements were taken from distinct samples or whether the same sample was measured repeatedly
☐	☒	The statistical test(s) used AND whether they are one- or two-sided<br>*Only common tests should be described solely by name; describe more complex techniques in the Methods section.*
☒	☐	A description of all covariates tested
☒	☐	A description of any assumptions or corrections, such as tests of normality and adjustment for multiple comparisons
☐	☒	A full description of the statistical parameters including central tendency (e.g. means) or other basic estimates (e.g. regression coefficient) AND variation (e.g. standard deviation) or associated estimates of uncertainty (e.g. confidence intervals)
☒	☐	For null hypothesis testing, the test statistic (e.g. *F*, *t*, *r*) with confidence intervals, effect sizes, degrees of freedom and *P* value noted<br>*Give P values as exact values whenever suitable.*
☒	☐	For Bayesian analysis, information on the choice of priors and Markov chain Monte Carlo settings
☒	☐	For hierarchical and complex designs, identification of the appropriate level for tests and full reporting of outcomes
☒	☐	Estimates of effect sizes (e.g. Cohen's *d*, Pearson's *r*), indicating how they were calculated

Our web collection on statistics for biologists contains articles on many of the points above.

Software and code

Policy information about availability of computer code

Data collection	Excel windows 10, GraphPad Prism8, Image Lab Software 6
Data analysis	Excel windows 10, GraphPad Prism8, Image Lab Software 6

For manuscripts utilizing custom algorithms or software that are central to the research but not yet described in published literature, software must be made available to editors/reviewers. We strongly encourage code deposition in a community repository (e.g. GitHub). See the Nature Research guidelines for submitting code & software for further information.

Data

Policy information about availability of data

All manuscripts must include a data availability statement. This statement should provide the following information, where applicable:

- Accession codes, unique identifiers, or web links for publicly available datasets
- A list of figures that have associated raw data
- A description of any restrictions on data availability

The data that support the findings of this study are available from the corresponding author upon reasonable request. Data can also be found in the supplementary data information file.

Field-specific reporting

Please select the one below that is the best fit for your research. If you are not sure, read the appropriate sections before making your selection.

☒ Life sciences ☐ Behavioural & social sciences ☐ Ecological, evolutionary & environmental sciences

Life sciences study design

All studies must disclose on these points even when the disclosure is negative.

Sample size	Group-size estimations were based upon a power calculation to minimally yield an 80% chance to detect a significant difference of P<0.05 between groups.
Data exclusions	There was no data exclusion.
Replication	In vivo studies were repeated in at least two different cohorts of mice. The findings were reproducable.
Randomization	All male littermates were used for the study. For aging experiments mice were randomly selected.
Blinding	For analyses of tissue samples, we used ascening numbers without knowing which sample belongs to which genotype.<br>However, for in vivo studies of middle-aged and aged mice blinding was not possible due to an obvious body weight phenotype.

Reporting for specific materials, systems and methods

We require information from authors about some types of materials, experimental systems and methods used in many studies. Here, indicate whether each material, system or method listed is relevant to your study. If you are not sure if a list item applies to your research, read the appropriate section before selecting a response.

Materials & experimental systems

n/a	Involved in the study	
☐	☒	Antibodies
☒	☐	Eukaryotic cell lines
☒	☐	Palaeontology
☐	☒	Animals and other organisms
☒	☐	Human research participants
☒	☐	Clinical data

Methods

n/a	Involved in the study	
☒	☐	ChIP-seq
☒	☐	Flow cytometry
☒	☐	MRI-based neuroimaging

Antibodies

Antibodies used

ABHD5 (Alpha/beta hydrolase domain containing 5) Abnova, Cat# H00051099-M01, clone 1F3, lot# n/a
ACC (Acetyl-coenzyme A carboxylase) Cell Signaling, Cat# 3676, clone C83B10, lot# n/a
AKT (RAC-alpha serine/threonine-protein kinase) Cell Signaling, Cat# 4691, clone C67E7, lot# n/a
ATGL (Adipose triglyceride lipase) Cell signaling, Cat# 2138S, lot# n/a
FAS (Fatty acid synthase) Cell Signaling, Cat# 3180, clone C20G5, lot# n/a
GAPDH (Glyceraldehyde-3-phosphate dehydrogenase) Cell signaling, Cat# 2118S, clone 14C10, lot# n/a
HSL (Hormone-sensitive lipase) Cell signaling, cat# 4107S, lot# n/a
PLIN1 (Perilipin-1) Sigma-Aldrich, Cat# P1998, lot# n/a
pAKT (Ser473) (RAC-alpha serine/threonine-protein kinase) Cell Signaling, cat# 9271S, lot# n/a
SCD1 (Stearoyl-CoA desaturase) Cell Signaling, cat# 2438, lot# n/a
SREBP1c (Sterol regulatory element-binding protein 1) Abcam, cat# ab3259, clone 2A4, lot# n/a

Validation

ABHD5 Western Blot knockout validation: PMID: 27559856, https://www.labome.com/knockout-validated-antibodies/ABHD5-antibody-knockout-validation-Abnova-H00051099-M01.html#ref1;
ATGL Western Blot knockout validation: PMID: 28988821, Cold-Induced Thermogenesis Depends on ATGL-Mediated Lipolysis in Cardiac Muscle, but Not Brown Adipose Tissue. (https://antibodyregistry.org/search.php?q=AB_2167955)
ACC, AKT, FAS, GAPDH validation orthogonal: statement on the manufacturer's website;
HSL Western blot knockout validation: in this manuscript;
PLIN1 Western blot overexpression validation and Immunocytochemistry knockout validation: PMID:25458893, Perilipin A is essential for the translocation of hormone-sensitive lipase during lipolytic activation, statement on the manufacturer's website.
SREBP1c validated in Western blot and tested in human: statement on the manufacturer's website.
pAKT, SCD1: validation unknown or not available; statement on the manufacturer's website or antibody database

Animals and other organisms

Policy information about studies involving animals; ARRIVE guidelines recommended for reporting animal research

Laboratory animals	We used mice for this study. We generated adipocyte-specific HSL knockout (AHKO) mice by crossing HSL-flox (HSLflox/flox) mice with mice expressing Cre recombinase under the control of the Adiponectin promoter.<br>Mice were backcrossed onto C57BL/6J for >N10.<br>For validation of the mouse model, we used male and female mice at an age from 12 weeks to 33 weeks, as stated in the manuscript.<br>For the charaterization, we used male mice at an age from 10 weeks to 44 weeks, as stated in the manuscript.
Wild animals	The study did not involve wild animals
Field-collected samples	The study did not involve samples collected from the field
Ethics oversight	All animal experiments were approved by the Austrian Federal Ministry for Science, Research, and Economy (protocol number BMWFW-66.007/0026/-WF/V/3b/2017) and the ethics committee of the University of Graz, and were conducted in compliance with the council of Europe Convention (ETS 123).

Note that full information on the approval of the study protocol must also be provided in the manuscript.
